# SPHK1 enhances olaparib resistance in ovarian cancer through the NFκB/NRF2/ferroptosis pathway

**DOI:** 10.1038/s41420-025-02309-y

**Published:** 2025-01-28

**Authors:** Kai Teng, Hanlin Ma, Panpan Gai, Xuelian Zhao, Gonghua Qi

**Affiliations:** 1 Department of Radiation Oncology, Shandong Cancer Hospital and Institute, Shandong First Medical University and Shandong Academy of Medical Sciences, Jinan, China; 2 Department of Obstetrics and Gynecology, Qilu Hospital, Shandong University, Jinan, China; 3 Gynecologic Oncology Key Laboratory of Shandong Province, Qilu Hospital, Shandong University, Jinan, China; 4 71217 of the Chinese People’s Liberation Army, Laiyang, China; 5 Department of Obstetrics and Gynaecology, People’s Hospital of Qihe County, Dezhou, China

**Keywords:** Ovarian cancer, Cancer therapeutic resistance

## Abstract

PARPis resistance is a challenge in the treatment of ovarian cancer. To investigate the potential mechanism involved in olaparib resistance of ovarian cancer, high-throughput sequencing was performed on olaparib-resistant SKOV3 cell line named SK/Ola. SPHK1 was upregulated in SK/Ola cells and was related to the PFS and OS in ovarian cancer patients. However, the effect and mechanism of SPHK1 on olaparib sensitivity in ovarian cancer were obscure. In this study, we found that SPHK1 promoted olaparib resistance. While, SPHK1 knockdown and SPHK1 inhibitor (PF-543 hydrochloride, named PF-543 in this article) enhanced the effect of olaparib on ovarian cancer cells. In mechanism, SPHK1 activated the NF-κB pathway through promoting p-IκBα degradation. Moreover, SPHK1 inhibited, but PF-543 activated ferroptosis in OC cells. Further investigation revealed that SPHK1 activated NF-κB p65, which in turn transcriptionally regulated NRF2 to inhibit ferroptosis in OC cells. Functionally, NF-κB p65 attenuated the PF-543-induced ferroptosis, and this effect was rescued by ferroptosis inducer erastin and RSL3. We conclude that SPHK1 inhibition triggers ferroptosis by restricting NF-κB-activated NRF2 transcription, thereby enhancing olaparib sensitivity in ovarian cancer. In vivo experiments also confirmed that the SPHK1 inhibitor increased olaparib sensitivity. A combination of SPHK1 inhibitors and olaparib may provide a therapeutic strategy for ovarian cancer.

## Introduction

Ovarian cancer (OC), one of the most common gynecological malignancies, has the worst prognosis and the highest mortality [1]. Poly (ADP-ribose) polymerase inhibitors (PARPis) significantly improve the progression-free survival (PFS) and overall survival (OS) of patients with OC, especially the one with homologous recombination deficiency (HRD) [1]. The US Food and Drug Administration (FDA) has approved PARPis (olaparib, niraparib, rucaparib, and talazoparib) to act as first-line and second-line maintenance treatment for OC patients [2, 3]. However, more than 40% of patients with BRCA1/2 deficiency do not respond to PARPis [4–6]. And, long-term use of PARPis often leads to acquired PARPis resistance [7]. Therefore, it is necessary to understand the mechanisms of PARPis resistance to enhance the sensitivity of PARPis.

To investigate the potential mechanism involved in olaparib resistance of ovarian cancer, we constructed an olaparib-resistant SKOV3 cell line named SK/Ola. Then, high-throughput sequencing was performed on the parental SKOV3 and SK/OLA cell lines, the results showed that sphingosine kinase 1 (SPHK1) was one of the upregulated genes and was related to the PFS and OS in ovarian cancer patients.

SPHK1 is an enzyme that catalyzes sphingosine phosphorylation to produce sphingosine-1-phosphate (S1P) [8]. SPHK1 has been reported to be upregulated and contributed to the survival, proliferation, migration, invasion and angiogenesis of various cancers [9–14] including ovarian cancer [15–17]. Meanwhile, SPHK1 was involved in resistance to chemotherapy [18] and treatment against SPHK1 has been identified as effective anti-cancer treatment for a variety of cancers [19–29]. Therefore, inhibitors targeting SPHK1 have been developed and tested [24].

Previous studies found that SPHK1 is overexpressed [15] and promotes the proliferation [17, 30, 31], metastasis and angiogenesis [15, 16] of ovarian cancer. Moreover, a SPHK1 inhibitor named SKI-II improved the effect of curcumin on the growth inhibition effect of ovarian cancer [32]. Prachi Gupta et al. [33] reported that PF-543, another specific SPHK1 inhibitor, combined with anti-PD-1 therapy improved ovarian cancer patients survival. However, the effect and mechanism of SPHK1 on olaparib sensitivity in ovarian cancer were obscure.

## Results

### SPHK1 is a key gene involved in olaparib resistance of ovarian cancer

**Fig. 1:**
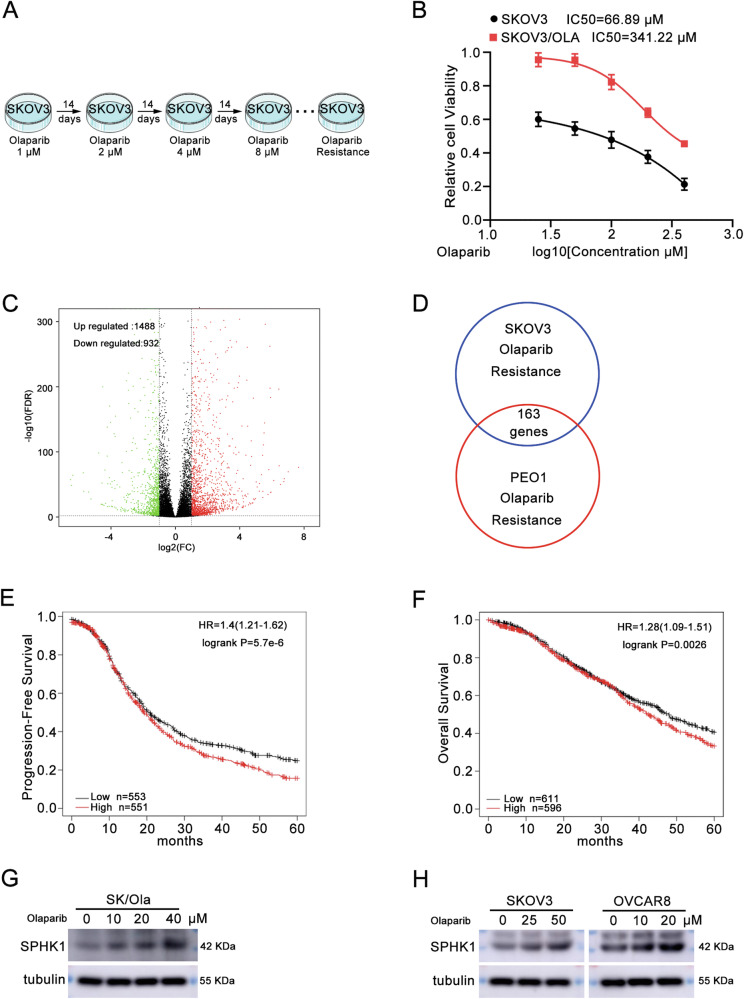
SPHK1 is a key gene involved in olaparib resistance of ovarian cancer. **A** The process of constructing an olaparib-resistant SKOV3 cell line. **B** The IC_50_ of olaparib in olaparib sensitive and resistant SKOV3 cells. **C** Volcano plot of RNA-sequencing data for olaparib-resistant SKOV3 cell line. **D** Upregulated differentially expressed genes in the RNA-sequencing data of olaparib-resistant SKOV3 and PEO1 cells. **E**, **F** The PFS (**E**) and OS (**F**) were analyzed based on SPHK1 expression by the K-M plotter database. **G** The protein level of SPHK1 in olaparib-resistant SKOV3 cells. **H** The protein level of SPHK1 in SKOV3 and OVCAR8 cells following olaparib treatment (*n* = 3).

To investigate the potential mechanism involved in olaparib resistance of ovarian cancer, we constructed an olaparib-resistant SKOV3 cell line named SK/Ola (Fig. 1A, B). Then, high-throughput sequencing was performed on the parental SKOV3 and SK/OLA cell lines; the results showed that 1488 genes were upregulated and 932 genes were downregulated (Fig. 1C). Furthermore, differentially expressed genes (DEGs) of olaparib-resistant PEO1 cell lines were downloaded and analyzed from the GEO database (GSE117765). Venny diagrams showed that 163 DEGs are jointly highly expressed in these two olaparib-resistant cell lines (Fig. 1D). Survival analysis showed that SPHK1 was related to the PFS and OS of ovarian cancer patients (Fig. 1E, F). Subsequently, we found that the SPHK1 level was significantly increased in SK/Ola (Fig. 1G, Original western blot Fig. 1A). Similarly, olaparib treatment increased the level of SPHK1 in SKOV3 and OVCAR8 cells (Fig. 1H, Original western blot Fig. 1B). These results indicated that SPHK1 might be a key gene leading to resistance to olaparib in ovarian cancer.

### SPHK1 promotes olaparib resistance in ovarian cancer cells

**Fig. 2:**
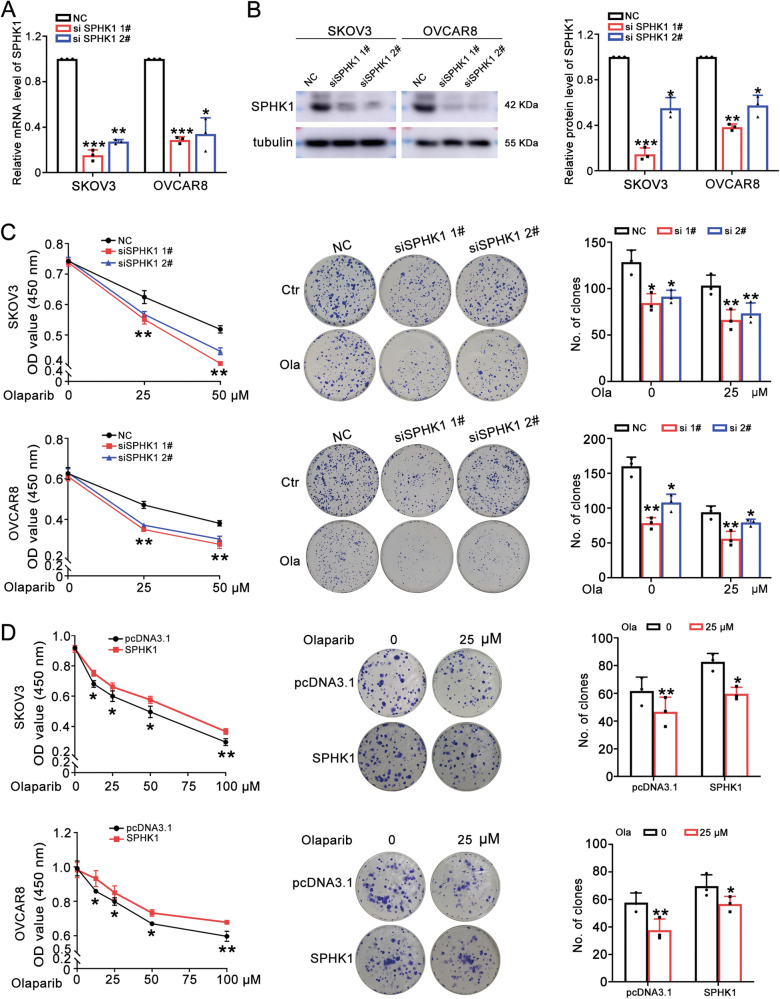
SPHK1 promotes olaparib resistance in OC cells. **A**, **B** The mRNA and protein level of SPHK1 in SKOV3 and OVCAR8 cells after SPHK1 knockdown. **C**, **D** The effect of SPHK1 knockdown and overexpression on the sensitivity to olaparib in SKOV3 and OVCAR8 cells were detected by MTT and colony survival assay (one-way ANOVA, Brown-Forsythe and Welch ANOVA tests, *p < 0.05, **p < 0.01, ***p < 0.001, mean ± SD, *n* = 3).

To research the role of SPHK1 in promoting olaparib resistance of ovarian cancer, we knocked down SPHK1 in SKOV3 and OVCAR8 cells (Fig. 2A, B and Original western blot Fig. 1C). Then, SPHK1 knockdown and the control OC cells were treated with gradient concentrations of olaparib. MTT and colony survival assays showed that SPHK1 knockdown ovarian cancer cells were more sensitive to olaparib (Fig. 2C). However, SPHK1 overexpressed SKOV3 and OVCAR8 cells exhibited stronger resistance to olaparib compared to the control group (Fig. 2D). Moreover, the cell death assay demonstrated that knockdown of SPHK1 increased the number of dead cells induced by olaparib, while overexpression of SPHK1 decreased this effect (Supplementary Fig. 1A,B). Taken together, SPHK1 promoted olaparib resistance and SPHK1 knockdown increased the sensitivity to olaparib of OC cells.

### PF-543, a SPHK1 inhibitor enhances the sensitivity of OC cells to olaparib

**Fig. 3:**
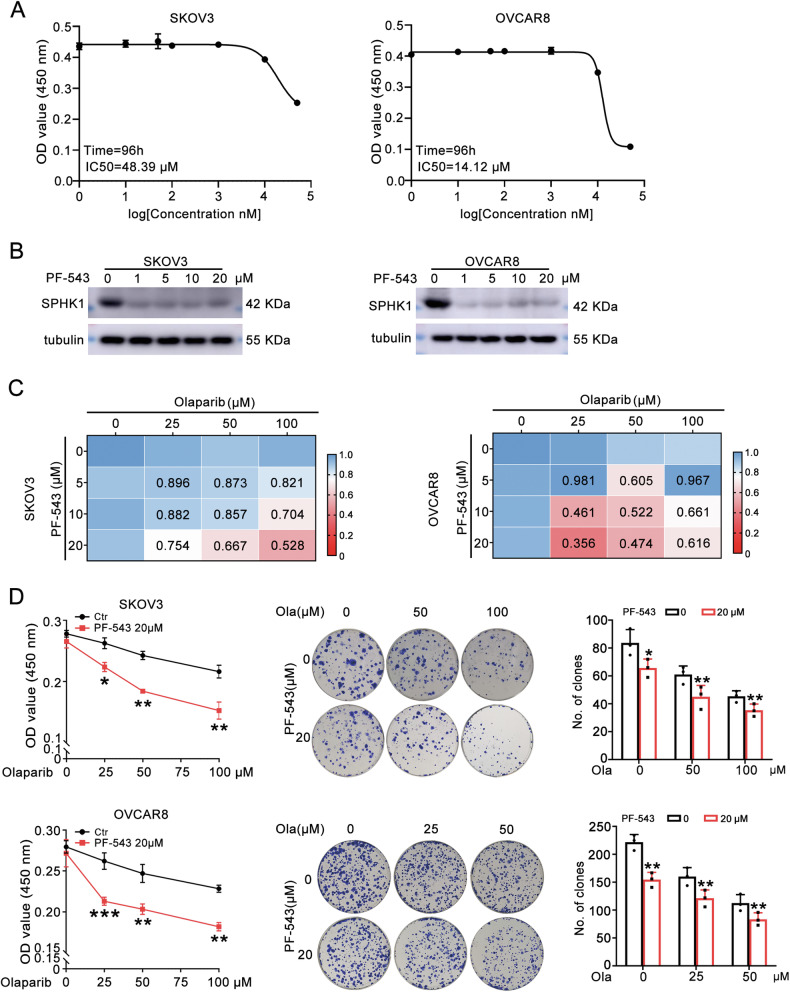
PF-543, a SPHK1 inhibitor enhances the sensitivity of OC cells to olaparib. **A** The IC_50_ of PF-543 in SKOV3 (IC_50_ = 48.39 μM, 96 h) and OVCAR8 (IC_50_ = 14.12 μM, 96 h) cells. **B** The protein of SPHK1 in SKOV3 and OVCAR8 cells following PF-543 treatment. **C** Combination Index of PF-543 and olaparib in SKOV3 and OVCAR8 cells were calculated by CompuSyn. **D** MTT and colony survival assays were used to evaluate the effect of PF-543 on the sensitivity to olaparib in SKOV3 and OVCAR8 cells.(one-way ANOVA, Brown-Forsythe and Welch ANOVA tests,*p < 0.05, **p < 0.01, ***p < 0.001, mean ± SD, *n* = 3).

PF-543, a SPHK1 inhibitor, was used to validate the role of SPHK1 in olaparib resistance of ovarian cancer. First, the IC_50_ of PF-543 in OC cells was calculated by MTT assay. The IC_50_ of PF-543 in SKOV3 and OVCAR8 cells was 48.39 μM and 14.12 μM, respectively (Fig. 3A, B and Original western blot Fig. 1D). Then, olaparib (0, 25, 50, 100 μM) and PF-543 (0, 5, 10, 20 μM) were used in combination and the synergistic antiproliferative effect was calculated. In SKOV3 cells, 100 µM olaparib plus 20 µM PF-543 had the strongest synergistic effect. While 25 µM olaparib plus 20 µM PF-543 had the most powerful effect in OVCAR8 cells (Fig. 3C). Moreover, MTT and colony survival assays showed that 20 μM PF-543 obviously increased sensitivity of ovarian cancer cells to olaparib (Fig. 3D). Additionally, PF-543 enhanced olaparib-induced cell death in SKOV3 and OVCAR8 cells (Supplementary Fig. 2). Collectively, SPHK1 inhibitor enhanced the effect of olaparib on OC cells.

### SPHK1 activates the NF-κB signaling pathway

**Fig. 4:**
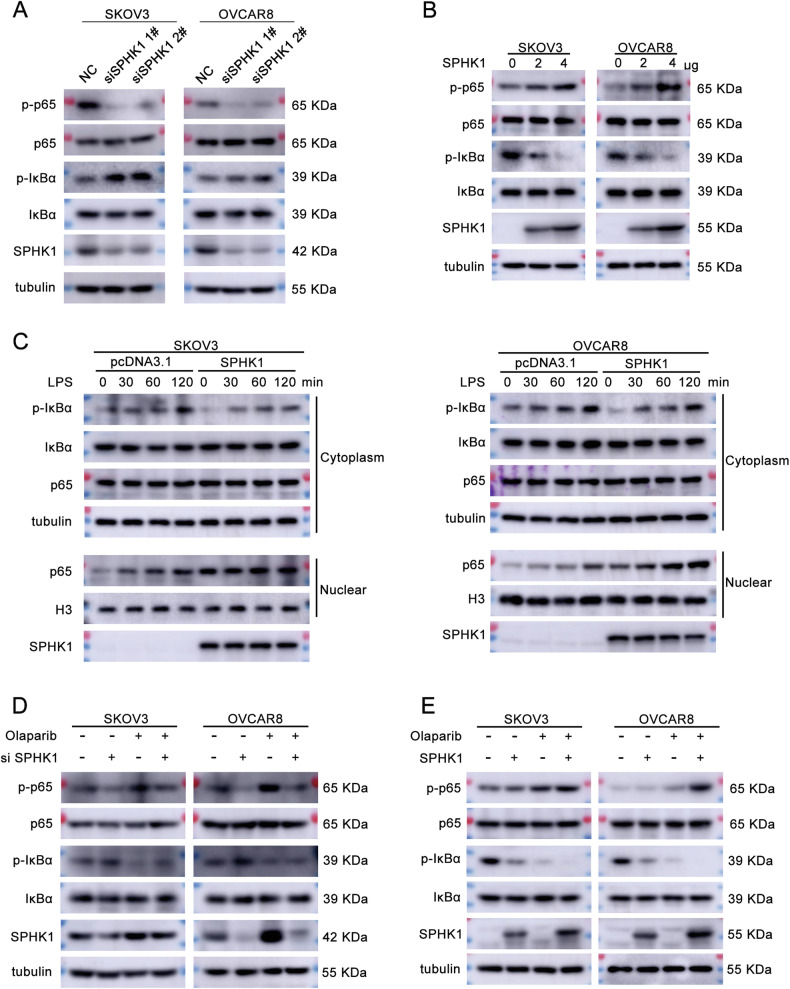
SPHK1 activates the NF-κB signaling pathway. **A**, **B** Protein levels of p-p65, p65, p-IκBα, IκBα, SPHK1 and tubulin in SPHK1 knockdown and overexpression cells were detected by western blotting assay. **C** SPHK1 overexpressed SKOV3 and OVCAR8 cells were treated with LPS (0, 30, 60, or 120 min), then the nuclear translocation of p65 and other NF-κB markers were assessed by western blotting. Tubulin and H3 were used as the cytoplasmic and nuclear internal control, respectively. **D** SKOV3 and OVCAR8 cells with SPHK1 knockdown were treated with or without olaparib, and a western blotting assay was performed to evaluate the levels of p-p65, p65, p-IκBα, IκBα, SPHK1, and tubulin. **E** SPHK1 overexpressed SKOV3 and OVCAR8 cells were treated with or without olaparib, and the levels of p-p65, p65, p-IκBα, IκBα, SPHK1, and tubulin were measured by a western blotting assay (*n* = 3).

To explore the potential mechanism that how SPHK1 promotes olaparib resistance in OC cells, SPHK1 knockdown OVCAR8 cells were conducted to high-throughput differential gene expression analysis. A total of 230 DEGs were detected, among which 99 are downregulated and 131 are overexpressed (Supplementary Fig. 3A). The KEGG analysis of the downregulated genes showed that the NF-κB signaling pathway was significantly inhibited (Supplementary Fig. 3B). Then, western blotting assay showed that SPHK1 knockdown decreased the level of p-p65 and increased the level of p-IκBα (Fig. 4A, Supplementary Fig. 4A and Original western blot Fig. 1E). SPHK1 overexpression showed the opposite result (Fig. 4B, Supplementary Fig. 4B and Original western blot Fig. 1F). Meanwhile, SPHK1 does not affect the levels of p65 and IκBα (Fig. 4A, B and Original western blot Fig. 1E, F). The degradation of p-IκBα and the nuclear translocation of p65 are key processes in the activation of the NF-κB signaling pathway. Therefore, we separated the nucleus and cytoplasm of LPS-treated cells and further detected the expression of p-IκBα in the cytoplasm and p65 in the nucleus. SPHK1 expression decreased the level of p-IκBα in the cytoplasm and increased the nuclear p65 level. At the same time, the expression levels of cytoplasmic IκBα and p65 did not show significant changes (Fig. 4C, Original western blot Fig. 2A). Furthermore, olaparib led to an increase in p-p65 levels and a decrease in p-IκBα levels. These effects of olaparib were reversed by SPHK1 knockdown, while overexpression of SPHK1 enhanced the effects of olaparib (Fig. 4D, E, Supplementary Fig. 4C, D and Original western blot Fig. 3A, B). From all the above, SPHK1 promoted olaparib resistance through the NF-κB signaling pathway.

### SPHK1 promotes the degradation of p-IκBα

**Fig. 5:**
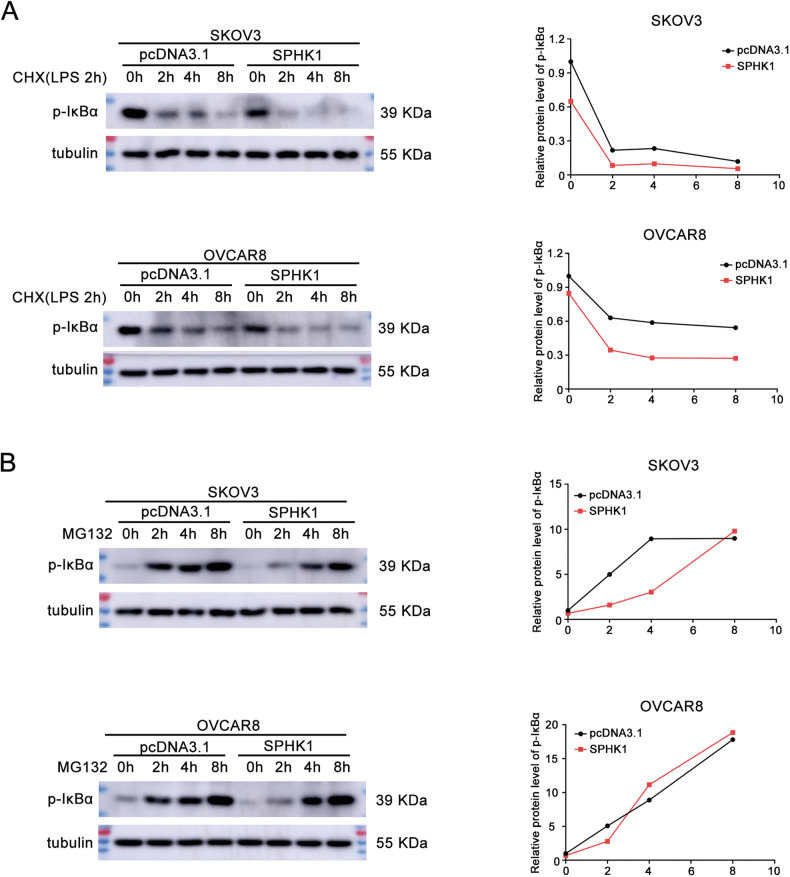
SPHK1 promotes the degradation of p-IκBα. **A** SPHK1 overexpressed SKOV3 and OVCAR8 cells were treated with LPS (1 μg/mL) for 2 h, then CHX (30 μM) was added for 0, 2, 4 and 8 h. Western blotting was used to detect the level of p-IκBα and tubulin. **B** SPHK1 overexpressed SKOV3 and OVCAR8 cells were treated with MG132 (10 μM) for different time. The level of p-IκBα and tubulin was detected by western blot (*n* = 3).

To determine whether SPHK1 promotes the degradation of p-IκBα, we utilized the protein synthesis inhibitor cycloheximide (CHX) and the proteasome inhibitor MG132. Cells were pretreated with LPS for 2 h, then CHX was added for 0, 2, 4 and 8 h. After CHX treatment, the half-life of p-IκBα in cells overexpressed SPHK1 is significantly shorter than that of control cells (Fig. 5A, Original western blot Fig. 4A). In SKOV3 cells, the level of p-IκBα reached similar levels in both groups approximately 8 h after MG132 treatment (Fig. 5B, Original western blot Fig. 4B). For OVCAR8 cells, the time at which the levels of p-IκBα became similar in SPHK1 overexpression and control groups was 4 h (Fig. 5B, Original western blot Fig. 4B). In conclusion, SPHK1 facilitates the degradation of p-IκBα to activate NF-κB signaling pathway.

### SPHK1 inhibits ferroptosis in ovarian cancer cells

**Fig. 6:**
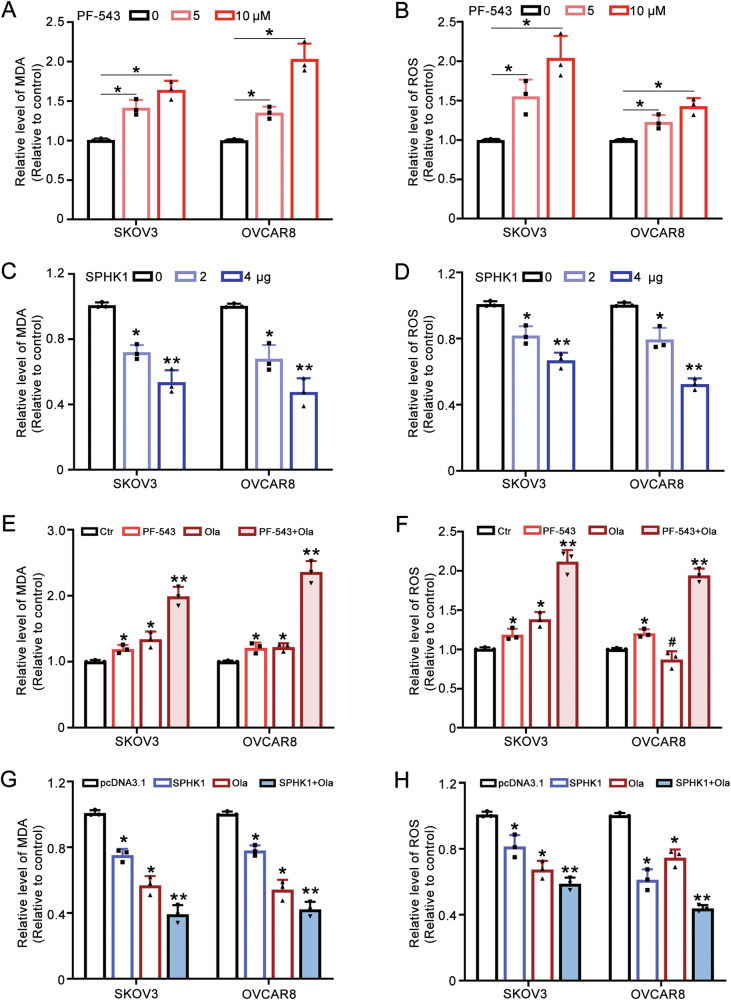
SPHK1 inhibits ferroptosis in ovarian cancer cells. **A** The level of MDA in PF-543 treated cells. **B** ROS assay was used to detect the level of ROS in PF-543-treated SKOV3 and OVCAR8 cells. **C**, **D** MDA and ROS in SPHK1 overexpressed cells were measured. **E**, **F** MDA and ROS assays were used to determine the effects of PF-543 and olaparib on ferroptosis, both individually and in combination. **G**, **H** SKOV3 and OVCAR8 cells overexpressing SPHK1 were treated with or without olaparib. Subsequently, MDA and ROS assays were conducted to detect the levels of MDA and ROS (one-way ANOVA, Brown-Forsythe and Welch ANOVA tests, *p < 0.05, **p < 0.01, mean ± SD, *n* = 3).

We have found that SPHK1 activates the NF-κB signaling pathway. Growing evidence has suggested that NF-κB contributes to the regulation of ferroptosis [34, 35]. Our attention has been drawn to whether SPHK1 is involved in the regulation of ferroptosis. PF-543 treatment led to the accumulation of MDA and lipid ROS (Fig. 6A, B) while SPHK1 overexpression decreased the level of MDA and ROS in OC cells (Fig. 6C, D). Furthermore, the combination of PF-543 and olaparib resulted in a more pronounced increase in MDA and ROS levels (Fig. 6E, F). However, the combination of SPHK1 with olaparib reduced the levels of MDA and ROS (Fig. 6G, H). Thus, SPHK1 inhibited, but PF-543 activated ferroptosis in OC cells.

### SPHK1 regulates NRF2 through p65 to inhibit ferroptosis in ovarian cancer cells

**Fig. 7:**
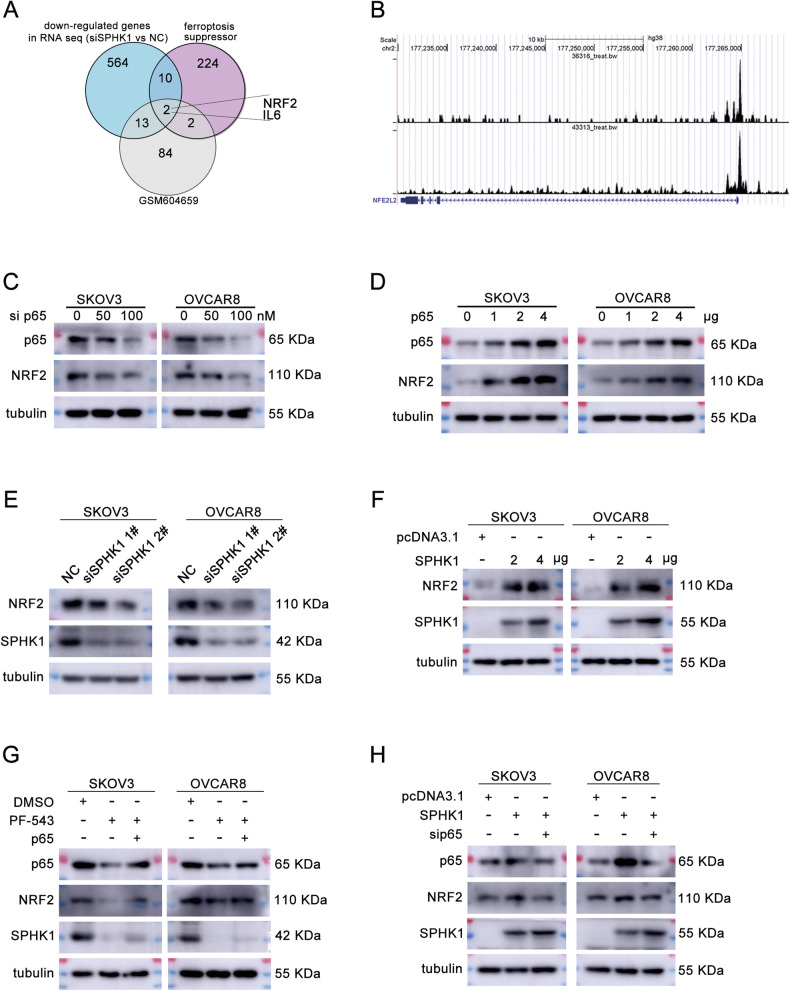
SPHK1 regulates NRF2 through P65 to inhibit ferroptosis in ovarian cancer cells. **A** A Venn diagram was created using RNA-seq data, ferroptosis suppressor data, and GSM604659 to visualize the overlaps among these datasets. **B** ChIP-seq data from the Cistrome Data Brower showed that NF-κB p65 binded to the promoter region of NRF2. **C** The protein levels of NRF2, p65 and tubulin in SKOV3 and OVCAR8 cells with p65 knockdown were detected by western blotting. **D** The levels of NRF2, p65 and tubulin were analyzed in SKOV3 and OVCAR8 cells transfected with p65 plasmid. **E** The protein levels of NRF2, SPHK1 and tubulin in SPHK1 knockdown SKOV3 and OVCAR8 cells were evaluated by western blotting. **F** Western blot assay was performed to measure the levels of NRF2, SPHK1 and tubulin in SKOV3 and OVCAR8 cells transfected with SPHK1 plasmid. **G** The level of p65, NRF2, SPHK1 and tubulin in SKOV3 and OVCAR8 cells treated with DMSO, PF-543, p65, and p65 combined with PF-543 were detected by western blotting. **H** SKOV3 and OVCAR8 cells overexpressing SPHK1 were subjected to p65 knockdown, and a western blot assay was conducted to examine the levels of p65, NRF2, SPHK1, and tubulin (*n* = 3).

To investigate whether SPHK1 inhibited ferroptosis through p65 and to clarify the potential mechanism. We compared the DEGs in SPHK1 knockdown group, ferroptosis suppressors and p65 targeted genes. Results showed that NRF2 and IL-6 were co-regulated genes (Fig. 7A). NRF2 has been illustrated to play an important role in mediating iron homeostasis and ferroptosis [36, 37]. Then, we analyzed ChIP-seq data of p65 from the Cistrome Data Browser database and found that p65 could bind to the promoter regions of NRF2 (Fig. 7B). Moreover, we found that NRF2 was downregulated by sip65 but upregulated by p65 in a dose-dependent manner in SKOV3 and OVCAR8 cells (Fig. 7C, D, Supplementary Fig. 5A, B, and Original western blot Fig. 5A, B). We further discovered that SPHK1 knockdown decreased and SPHK1 overexpression increased the level of NRF2 (Fig. 7E, F, Supplementary Fig. 5C, D and Original western blot Fig. 5C, D). What’s more, PF-543 downregulated the NRF2 level, which could be rescued by overexpression of p65 (Fig. 7G, Supplementary Fig. 6A and Original western blot Fig. 5E). Accordingly, the elevated NRF2 level resulted by SPHK1 could be rescued by sip65 (Fig. 7H, Supplementary Fig. 6B and Original western blot Fig. 5F). Collectively, SPHK1 inhibited ferroptosis through activating NF-κB p65 transcripted NRF2 in ovarian cancer cells.

### PF-543 enhances olaparib sensitivity of OC by inhibiting p65, which suppressed ferroptosis

**Fig. 8:**
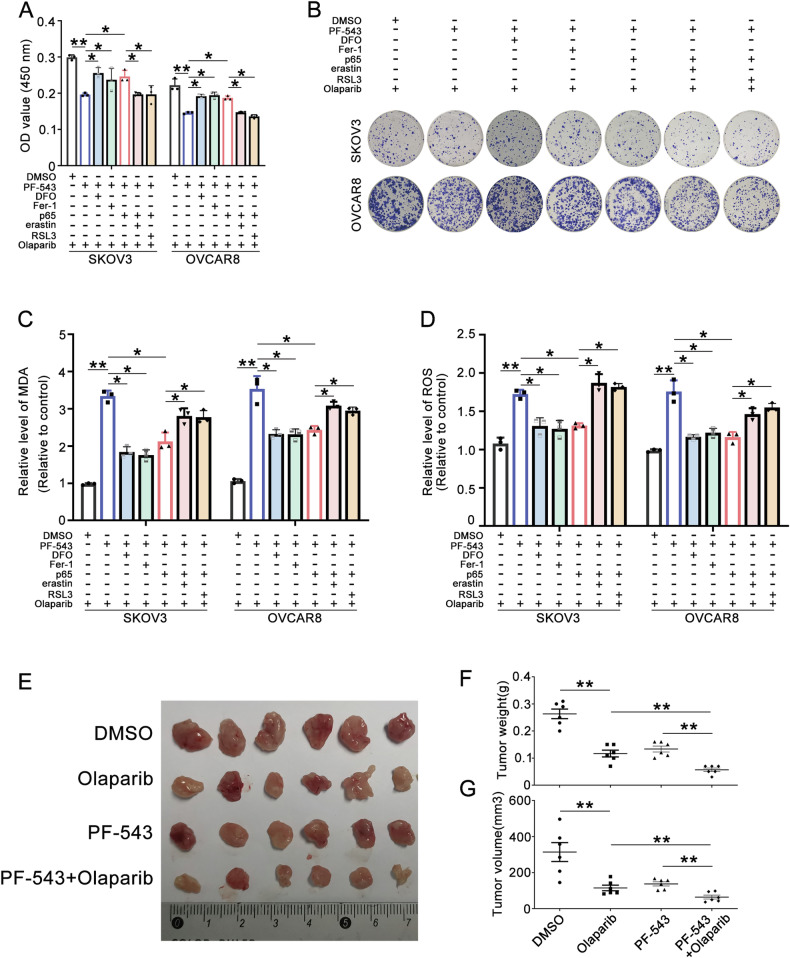
PF-543 enhances olaparib sensitivity of OC by inhibiting p65 suppressed ferroptosis. **A**–**D** SKOV3 and OVCAR8 cells were treated with olaparib or a combination of olaparib and PF-543 for 48 h. Subsequently, DFO, Fer-1, p65, erastin, and RSL3 were added as indicated in the figure for an additional 24 h. **A** MTT assay was used to determine cell viability. **B** Colony survival assays were performed to evaluate the effects of the indicated drugs on the olaparib sensitivity of SKOV3 and OVCAR8 cells. **C**, **D** MDA and ROS were measured. **G** The effects of DMSO, olaparib, PF-543, PF-543+olaparib on the growth of OVCAR8 cells were analyzed in a xenograft mouse model. **E** Representative tumor images. **F** Tumor weight of (**E**). **G** Tumor volume was shown (one-way ANOVA, Brown-Forsythe and Welch ANOVA tests, *p < 0.05, **p < 0.01, mean ± SD, *n* = 3).

We were concerned whether PF-543 could enhance the olaparib sensitivity of OC by p65-inhibited ferroptosis. First, the MTT and colony survival assays demonstrated that PF-543 increased sensitivity to olaparib, an effect that could be mitigated by the ferroptosis inhibitors DFO and Fer-1. Additionally, overexpression of p65 yielded similar results with DFO and Fer-1. Furthermore, erastin and RSL3 were found to counteract the effects caused by p65 (Fig. 8A, B). Furthermore, analyses using MDA and ROS assays indicated that p65 could attenuate PF-543-induced ferroptosis, producing effects similar to those of DFO and Fer-1. Conversely, erastin and RSL3 were observed to counteract the suppressive effect of p65 on ferroptosis induction (Fig. 8C, D). To better elucidate the effect of PF-543 on olaparib sensitivity in vivo, we subcutaneously injected the OVCAR8 cells into 5-week-old BALB/c athymic nude mice. The results indicated that both olaparib and PF-543 could inhibit tumor growth when used individually. Meanwhile, the inhibitory effects were more pronounced under the combined treatment of olaparib and PF-543 (Fig. 8E–G).

Therefore, we conclude that PF-543 enhanced the sensitivity of OC to olaparib by inhibiting ferroptosis which was suppressed by p65.

## Discussion

SPHK1 has been reported to contribute to chemotherapy resistance [38]. Therefore, SPHK1-specific inhibitors have been developed and identified to be effective treatment for various cancers [24]. SPHK1 was overexpressed in tissues and cells of ovarian cancer [15, 32]. The combination of a SPHK1 inhibitor named SKI-II or SPHK1 knockdown with curcumin has shown promising effects in enhancing growth inhibition and inducing apoptosis in ovarian cancer cells [32]. The study by Prachi Gupta et al. suggests that combining PF-543 with anti-PD-1 improves the survival of ovarian cancer patients [33]. However, the effect and mechanism of SPHK1 on olaparib sensitivity remain unclear. In this study, we found that SPHK1 promoted olaparib resistance. While, SPHK1 knockdown and SPHK1 inhibitor enhanced the effect of olaparib on OC cells.

RNA-seq was used to explore the potential mechanism that how SPHK1 promotes olaparib resistance in OC cells. The findings from the RNA-seq analysis indicate that SPHK1 may promote olaparib resistance in OC cells by inhibiting the NF-κB signaling pathway. It has been reported that SPHK1 promotes the progression of breast epithelial cells through NF-κB-p65 [39]. SPHK1 promoted the progression and metastasis of oral squamous cell carcinoma via NF-κB-p65 [40]. SPHK1 activated the PI3K/Akt/NF-kB pathway in non-small cell lung cancer [28]. Similarly, our results revealed that SPHK1 knockdown decreased and SPHK1 overexpression increased the level of p-p65. Furthermore, nucleus and cytoplasm separation showed that SPHK1 expression decreased the level of p-IκBα in the cytoplasm and increased the nuclear p65 level. The degradation of p-IκBα and the nuclear translocation of p65 are key processes in the activation of the NF-κB signaling pathway. It has been reported that SPHK1 and its product S1P are necessary for IκBα degradation, leading to NF-κB activation [41]. In this study, MG132 and CHX assay revealed that SPHK1 activates the NF-κB pathway by promoting p-IκBα degradation.

Growing evidence has suggested that NF-κB contributes to the regulation of ferroptosis [34, 35]. Therefore, whether SPHK1 is involved in the regulation of ferroptosis and whether it modulates ferroptosis through the NF-κB pathway are points of interest for further investigation. Results showed that SPHK1 inhibited and PF-543 activated ferroptosis in OC cells. Further investigation revealed that SPHK1 activated p65, which in turn transcriptionally regulates NRF2. Current evidence has demonstrated that there is cross-talk between NF-κB and NRF2 [42]. Recently, Rushworth et al. and Ziyuan Wang et al. proposed that NF-κB binds to the promoter region of NRF2, thereby positively regulating NRF2 promoter activity [43, 44]. NRF2 has been illustrated to play an important role in mediating iron homeostasis and ferroptosis [36, 37]. Besides, NRF2 regulates the expression of SLC7A11 and GPX4, both of which are ferroptosis inhibitors [37]. Thus, SPHK1 inhibits ferroptosis in ovarian cancer cells through P65 regulated NRF2.

In conclusion, we reveal that SPHK1 promoted olaparib resistance and SPHK1 inhibition enhanced the effect of olaparib on OC cells. SPHK1 activates NF-κB p65 through promoting p-IκBα degradation. Moreover, SPHK1 activated NF-κB p65 transcribed NRF2 to inhibit ferroptosis in OC cells. SPHK1 inhibition triggers ferroptosis by restricting NF-κB-activated NRF2 transcription, thereby enhancing olaparib sensitivity in ovarian cancer. In vivo experiments also confirmed that the SPHK1 inhibitor increased olaparib sensitivity. Future studies will incorporate patient-derived xenograft (PDX) models, organoids, and clinical data from ovarian cancer patients to further explore these findings.

## Materials and methods

### Cell lines and cell culture

SKOV3 cell line was from the American Type Culture Collection (ATCC, Manassas, VA, USA). Olaparib-resistant SKOV3 (SKOV3/Ola) cells were constructed by exposing parental SKOV3 cells to gradually increasing concentrations of olaparib in our lab. OVCAR8 was obtained from the M.D. Anderson Cancer Center characterized Cell line Core. SKOV3 and SKOV3/Ola cells were cultured in Dulbecco’s modified Eagle’s medium (DMEM) with 10% fetal bovine serum (FBS). OVCAR8 cells were maintained in RPMI 1640 with 10% FBS. The culture environment is a humidified atmosphere at 37 °C with 5% CO_2_. All cell lines have been authenticated by short tandem repeat (STR) profiling.

### Chemicals

Olaparib (S1060), PF-543 (S7177), CHX (S7218), MG132 (S2619) and erastin (S7242) were purchased from Selleck Chemicals (Houston, TX, USA). DFO (A427069), Fer-1 (A417669), RSL3 (A421791) were gained from Sangon Biotech (Shanghai, China).

### Stable and transient transfection

The SPHK1 overexpression plasmid (SPHK1) and the corresponding control vector pcDNA3.1 were obtained from YouBio (Hunan, China). Cells at appropriate confluence were transfected with SPHK1/pcDNA3.1 plasmid with Lipofectamin 2000 (11668-019, Invitrogen) according to the manufacturer’s instructions. Forty-eight hours after transfection, the cells were selected with corresponding medium in the presence of 500 ng/mL neomycin (G418 Sulfate, Sangon, Shanghai) for 2 weeks.

Ovarian cancer cells at an appropriate confluence were transfected with siSPHK1 or NC using Lipofectamine 2000 according to the manufacturer’s instructions (11668-019, Invitrogen). Specific SPHK1 siRNAs (siSPHK1 1#: 5’–GGGCAAGGCCUUGCAGCUCTT–3’; siSPHK1 2#: 5’–GGCUGAAAUCUCCUUCACGTT–3’) and a negative control siRNA (NC: 5’-UUCUCCGAACGUGUCACGUTT-3’) were purchased from GenePharma (Shanghai, China).

### Cytotoxicity analysis

MTT and colony survival assays were used to detect the effect of SPHK1 on ovarian cancer cells’ sensitivity to olaparib as described before [45]. The combination effect between olaparib and PF-543 was calculated by CompuSyn software with the ChouTalalay method. Combination indexes (CI) < 1 means synergistic effects.

### Cell death assay

SPHK1 knockdown or overexpression cells at 50% confluence were treated with olaparib for 48 h. Then, cells were collected and stained with FITC Annexin V and PI (556547, BD Bioscience, Franklin Lakes, NJ, USA) according to the manufacturer’s instructions. A flow cytometer (FACSCalibur, BD, USA) was used to capture the cells. The percentage of PI positivity was used as an indicator of cell death.

### RNA isolation and RT-qPCR

The procedure of RNA isolation and RT-qPCR was the same as recorded before [45]. The sequences for the primer pairs were as follows: SPHK1 forward primer: GCTGGCAGCTTCCTTGAACCAT; reverse primer: GTGTGCAGAGACAGCAGGTTCA; and β-actin forward primer: CACCATTGGCAATGAGCGGTTC; reverse primer: AGGTCTTTGCGGATGTCCACGT.

### Protein extraction and western blotting

Protein extraction and western blotting were performed as before [45]. Antibodies were as follows: SPHK1 (1:1000, Proteintech, 10670-1-AP), tubulin (1:5000, Proteintech, 11224-1-AP), NF-κB p65 (1:1000, CST, 8242T), p-NF-κB p65 (1:1000, CST, 3033T), IκBα (1:1000, CST, 4814T), p-IκBα (1:1000, CST, 2859T), H3 (1:5000, Proteintech, 17168-1-AP).

### Nuclear and cytoplasmic protein extraction

Nuclear and cytoplasmic protein extraction was performed by Nuclear and Cytoplasmic Extraction Kit (Beyotime, P0027, Shanghai, China) according to the manufacturer’s instructions.

### Bioinformatics analysis

Survival analysis based on SPHK1 expression was performed using the Kaplan–Meier plotter (http://kmplot.com/analysis/). GSE117765 contained differentially expressed genes (DEGs) of olaparib-resistant ovarian cancer cell lines downloaded from the GEO database. The DEGs were analyzed by GEO2R with log2 |fold change| > 1 and *p*-value < 0.05. Ferroptosis-related suppressors were downloaded from FerrDb. The JASPAR (http://jaspar.binf.ku.dk) website was used to search the potential targeted gene of NFκB p65.

### High-throughput differential gene expression analysis

Parental SKOV3 and SKOV3/Ola cell lines were used to analyze the DEGs involved in olaparib resistance of ovarian cancer and the analysis was performed by BioMarker Technologies (Beijing, China). DEGs and KEGG analysis between SPHK1 knockdown (siSPHK1) and NC group (*n* = 3) was conducted by BioMarker Technologies (Beijing, China). Total RNA was extracted from OVCAR8 cells 48 h after transfection with siSPHK1 or NC (*n* = 3). DEGs refer to genes with a |fold change| (FC) > 2 and an adjusted *p*-value (FDR) < 0.05.

### Malondialdehyde (MDA) assays

Lipid peroxidation MDA assay kit (Beyotime, S0131S, Shanghai, China) was applied to measure the relative MDA concentration according to the manufacturer’s protocol. The cells were lysed, centrifuged, the supernatant was collected and the protein concentration in the supernatant was determined. 100 μL supernatant from each sample was moved to a new 1.5 mL centrifuge tube and 200 μL MDA working fluid was added. Then the samples were mixed and incubated at 100 °C for 15 min. Next, 200 μL reaction mixture was transferred to a 96-well plate, and the absorbance was measured at 532 nm after the reaction mixture was cooled to room temperature.

### Lipid ROS assays

Reactive Oxygen Species Assay Kit (Beyotime, S0033S, Shanghai, China) was used to detect intracellular ROS level. Approximately 3000 cells were seeded to each well of 96-well plates and treated with indicated drugs for 48 h, then 10 μM DCFH-DA was added and incubated for another 30 min. After washing with PBS three times, the levels of lipid ROS were detected by the Varioskan Flash microplate reader (Thermo Scientific) with the excitation wavelength at 488 nm and the emission wavelength at 525 nm.

### Tumor formation assay in nude mice

The tumor formation assay was approved by the Shandong University Animal Care and Use Committee. Female athymic BALB/c nude mice about 5 weeks old (NBRI of Nanjing University, Nanjing, China) in a pathogen-free facility were used. Approximately 5 × 10^6^ OVCAR8 cells were resuspended with 150 μL PBS and subcutaneously injected into the left armpit of each mouse. The tumor volumes were observed every 2 days until the tumor volumes were about 50 mm^3^. Then, the mice were randomly divided into four subgroups and treated with DMSO, olaparib, PF-543, olaparib+PF-543. Olaparib (50 mg/kg) and PF-543 (10 mg/kg) were intraperitoneally injected every day separately or in combination. Fourteen days post-injection, the mice were sacrificed, and the tumors were separated.

### Statistical analysis

Data were presented as means ± SD and three repetitions were performed for each experiment. Statistical Product and Service Solutions (SPSS, Inc., Chicago, IL, USA) (22.0) statistical software was used to analyze the data. The Brown-Forsythe and Welch ANOVA tests in one-way ANOVA were employed to assess the significance across more than two groups (^#^*p* > 0.05, *p < 0.05, **p < 0.01, and ***p < 0.001). GraphPad Prism 8.00 (GraphPad Software, La Jolla, CA, USA) and Adobe Photoshop CC 2019 (Adobe, San Jose, CA, USA) were applied to dispose the images.

## Supplementary information

Supplementary Figures

Original figures of western blot

## Data Availability

The data that support the findings of this study are available from the corresponding author upon reasonable request.
