# Supplementary material for: SPHK1 enhances olaparib resistance in ovarian cancer through the NFκB/NRF2/ferroptosis pathway
Source: Cell Death Discov. 2025 Jan 28;11:29. doi: 10.1038/s41420-025-02309-y (PMC11775125; doi:10.1038/s41420-025-02309-y)
Supplement: Supplementary file 1 — Supplementary Figures [file 41420_2025_2309_MOESM1_ESM.docx]

**Supplementary figures and figure legends:**

**Supplementary figure legends：**

**Supplementary Figure 1. SPHK1 knockdown enhanced, while SPHK1 overexpression reduced, olaparib-induced cell death in OC cells.**

A and B. SKOV3 and OVCAR8 cells with either SPHK1 knockdown or overexpression were treated with olaparib for 48 hours. A cell death assay was conducted to quantify the number of dead cells. (one-way ANOVA, Brown-Forsythe and Welch ANOVA tests, **p* < 0.05, ***p* < 0.01, mean ± SD, n = 3 ).

**Supplementary Figure 2. PF-543 increased olaparib-induced cell death.**

SKOV3 and OVCAR8 cells were treated with DMSO, PF-543, olaparib, or a combination of PF-543 and olaparib. A flow cytometer was utilized to assess cell death. (one-way ANOVA, Brown-Forsythe and Welch ANOVA tests, **p* < 0.05, ***p* < 0.01, mean ± SD, n = 3 ).

**Supplementary Figure 3. NGS analysis of the signaling pathways affected by SPHK1 knockdown.**

Next-generation sequencing (NGS) was carried out in OVCAR8 cells transfected with siSPHK1 1# or NC (n=3) to reveal the mRNA expression profile.

A. Volcano plot displayed the DEGs between siSPHK1 and NC groups.

B. The KEGG pathway analysis of the down-regulated DEGs.

**Supplementary Figure 4. Quantification of Figure 4.**

1. Quantification of the protein levels in Figure 4A.
2. Quantification of the protein levels in Figure 4B.
3. Quantification of the protein levels in Figure 4D.
4. Quantification of the protein levels in Figure 4E.

(one-way ANOVA, Brown-Forsythe and Welch ANOVA tests, **p* < 0.05, ***p* < 0.01, mean ± SD, n = 3 ).

**Supplementary Figure 5. Quantification of Figure 7C-F.**

1. Quantification of the protein levels in Figure 7C.
2. Quantification of the protein levels in Figure 7D.
3. Quantification of the protein levels in Figure 7E.
4. Quantification of the protein levels in Figure 7F.

(one-way ANOVA, Brown-Forsythe and Welch ANOVA tests, **p* < 0.05, ***p* < 0.01, ****p* < 0.001, mean ± SD, n = 3 ).

**Supplementary Figure 6. Quantification of Figure 7G-H.**

1. Quantification of the protein levels in Figure 7G.
2. Quantification of the protein levels in Figure 7H.

(one-way ANOVA, Brown-Forsythe and Welch ANOVA tests, ^#^*p*＞0.05，**p* < 0.05, ***p* < 0.01, ****p* < 0.001, mean ± SD, n = 3 ).

**Supplementary Figure 1**

**
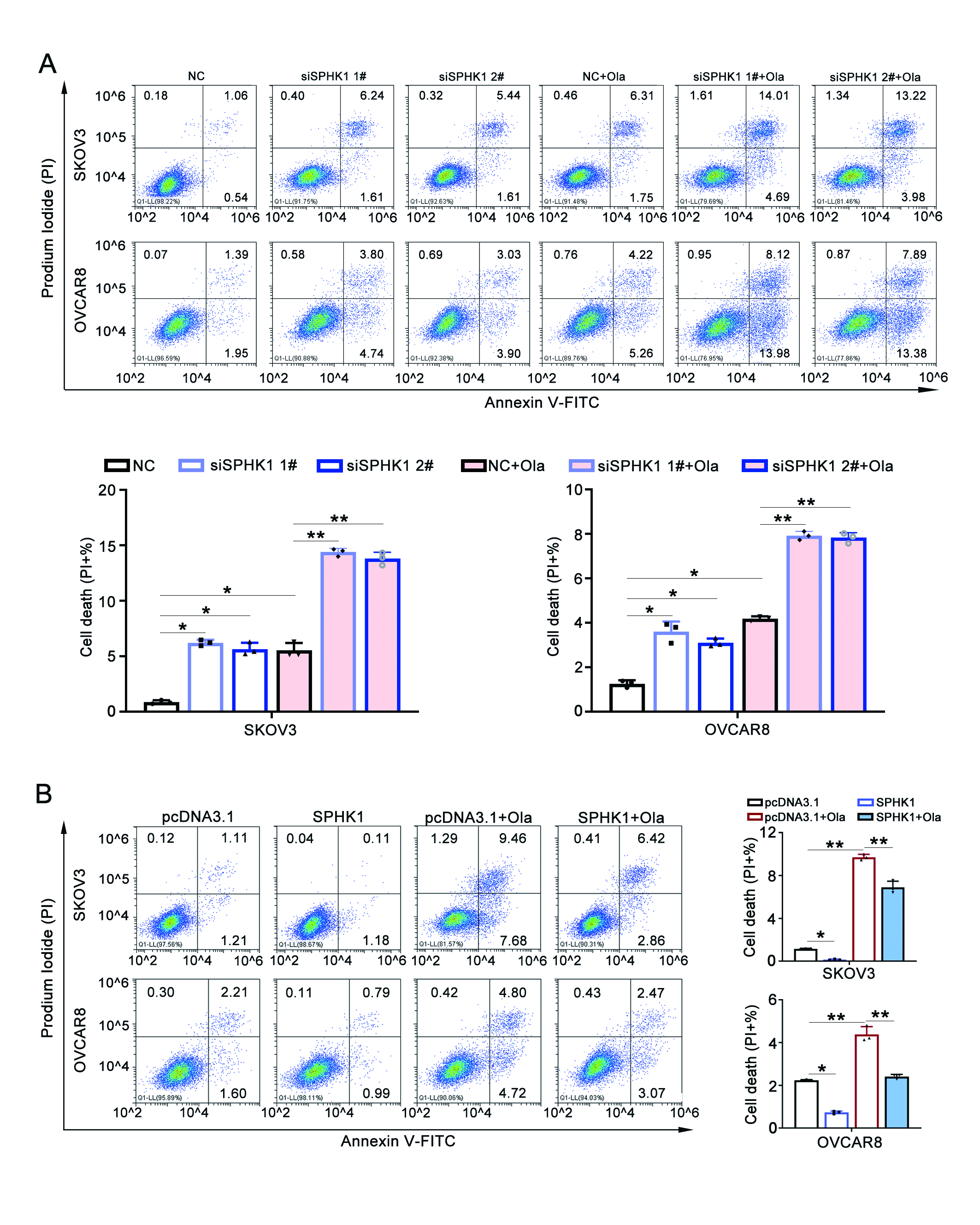
**

**Supplementary Figure 2**

**
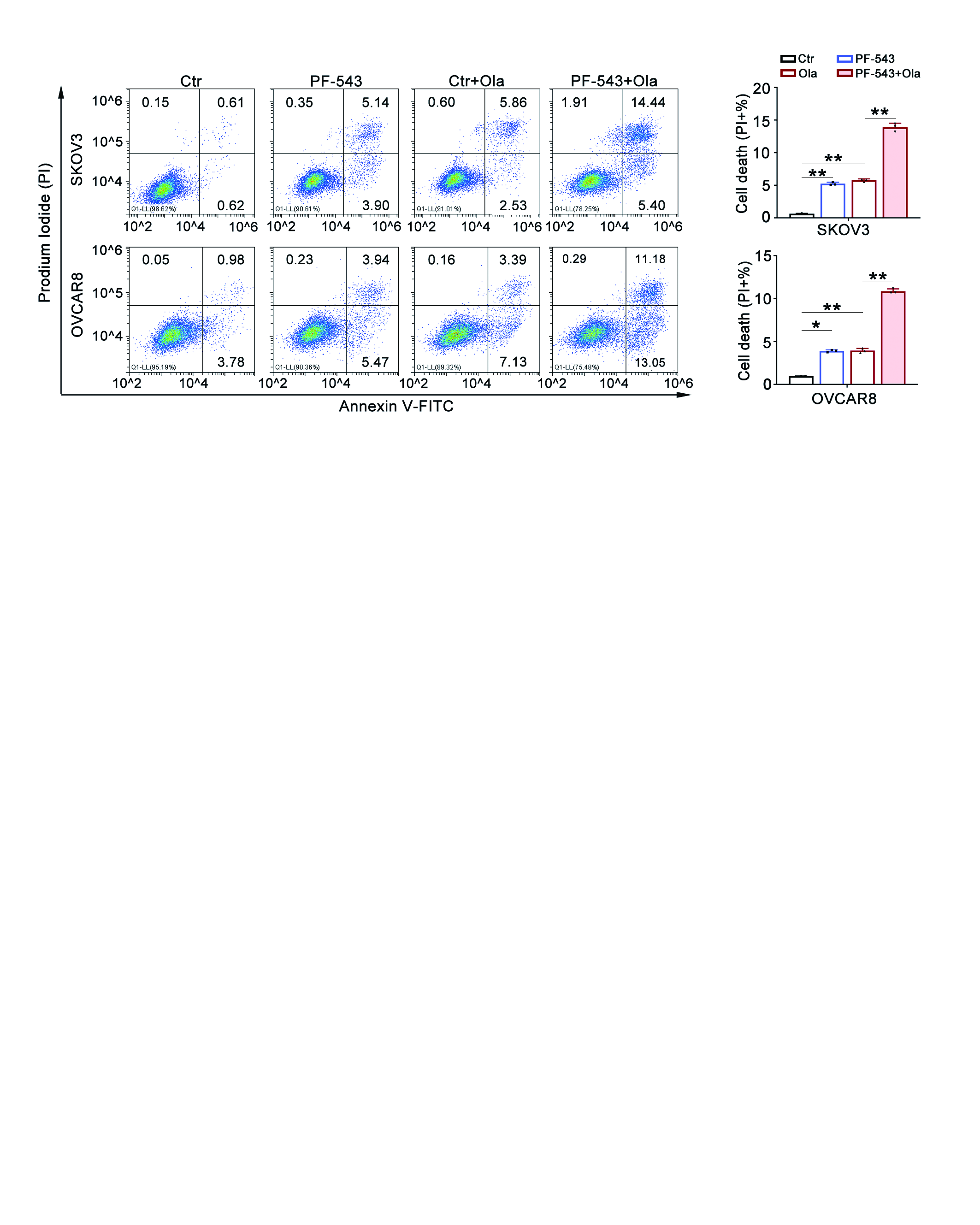
**

**Supplementary Figure 3**

**
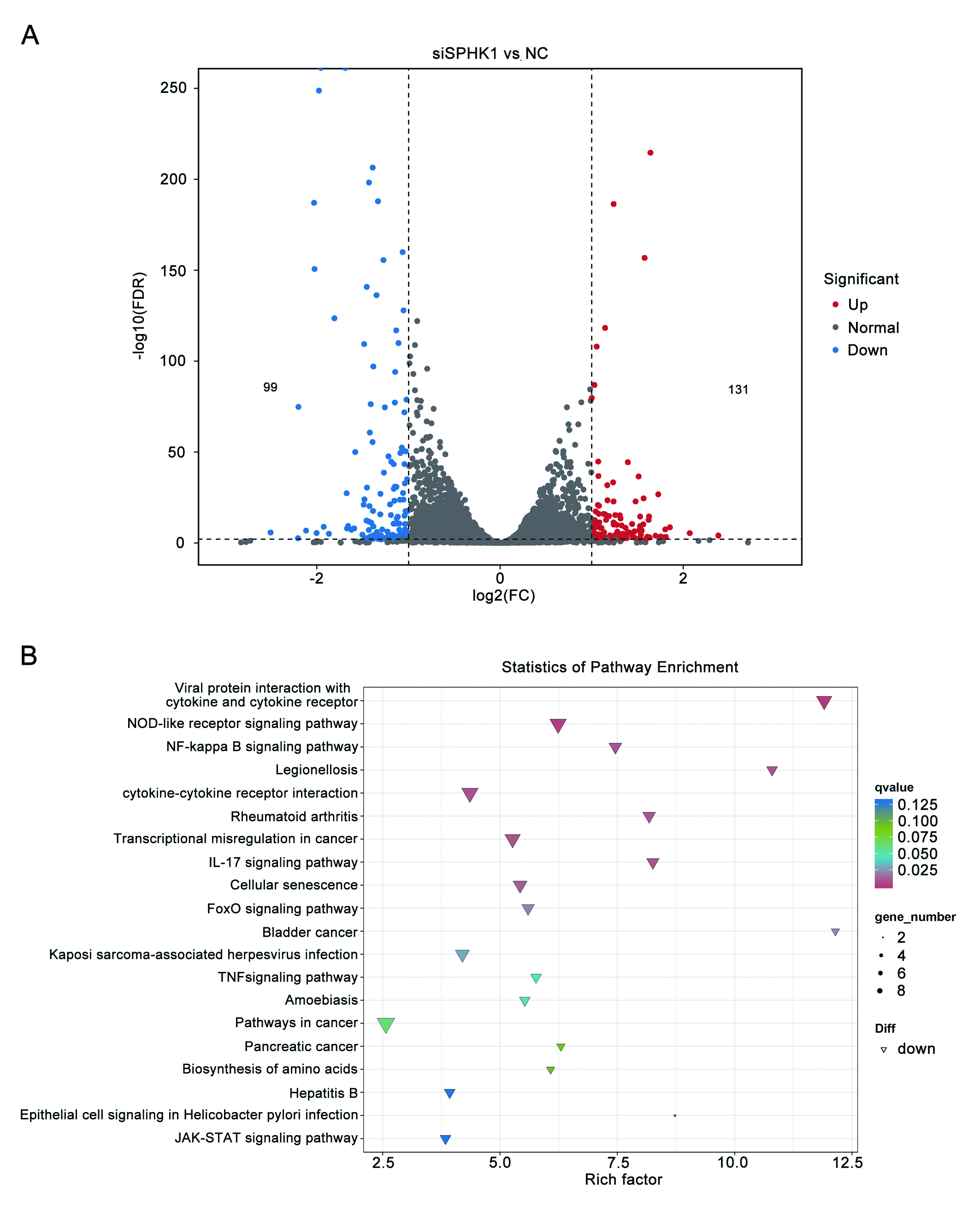
**

**Supplementary Figure 4**

**
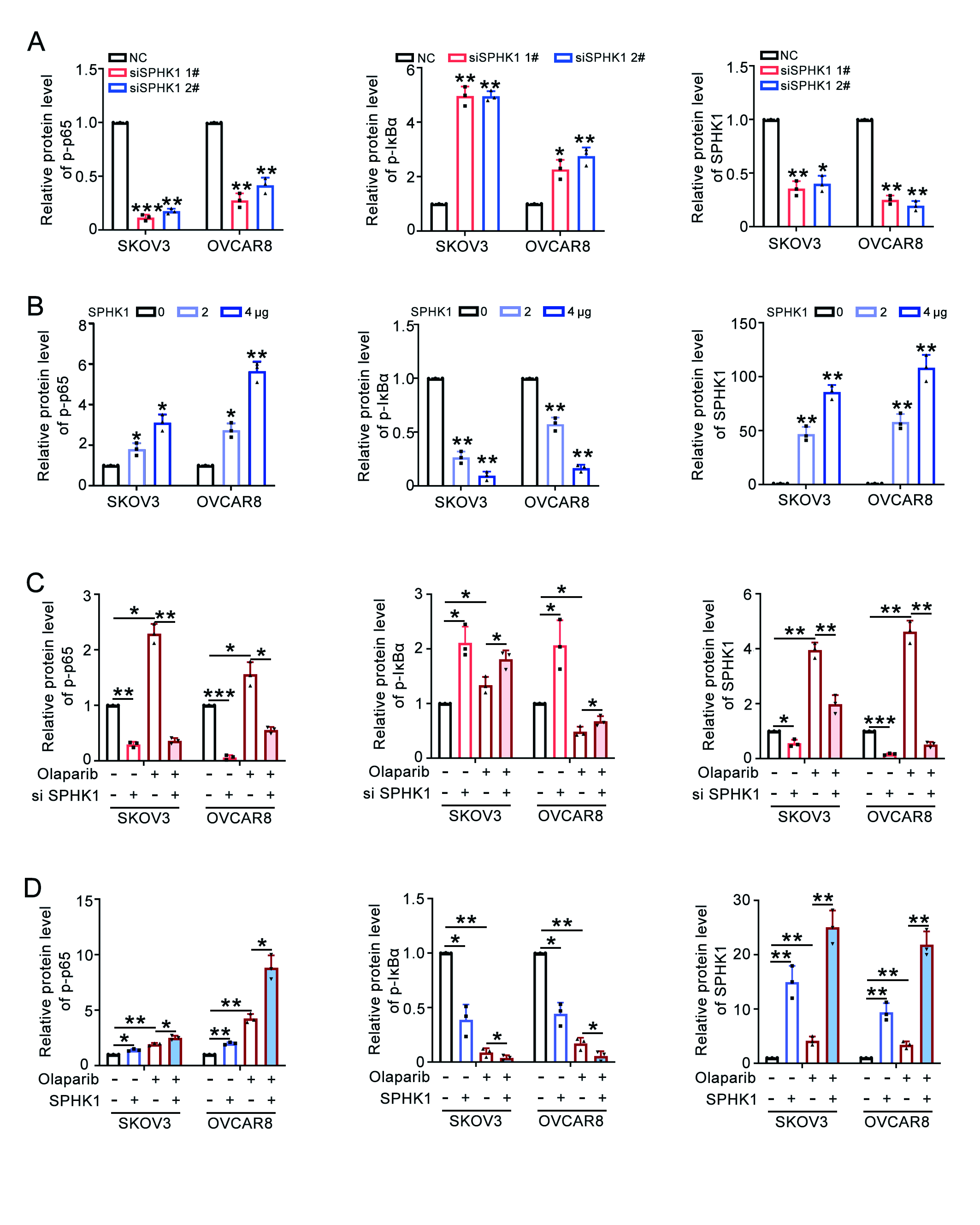
**

**Supplementary Figure 5**

**
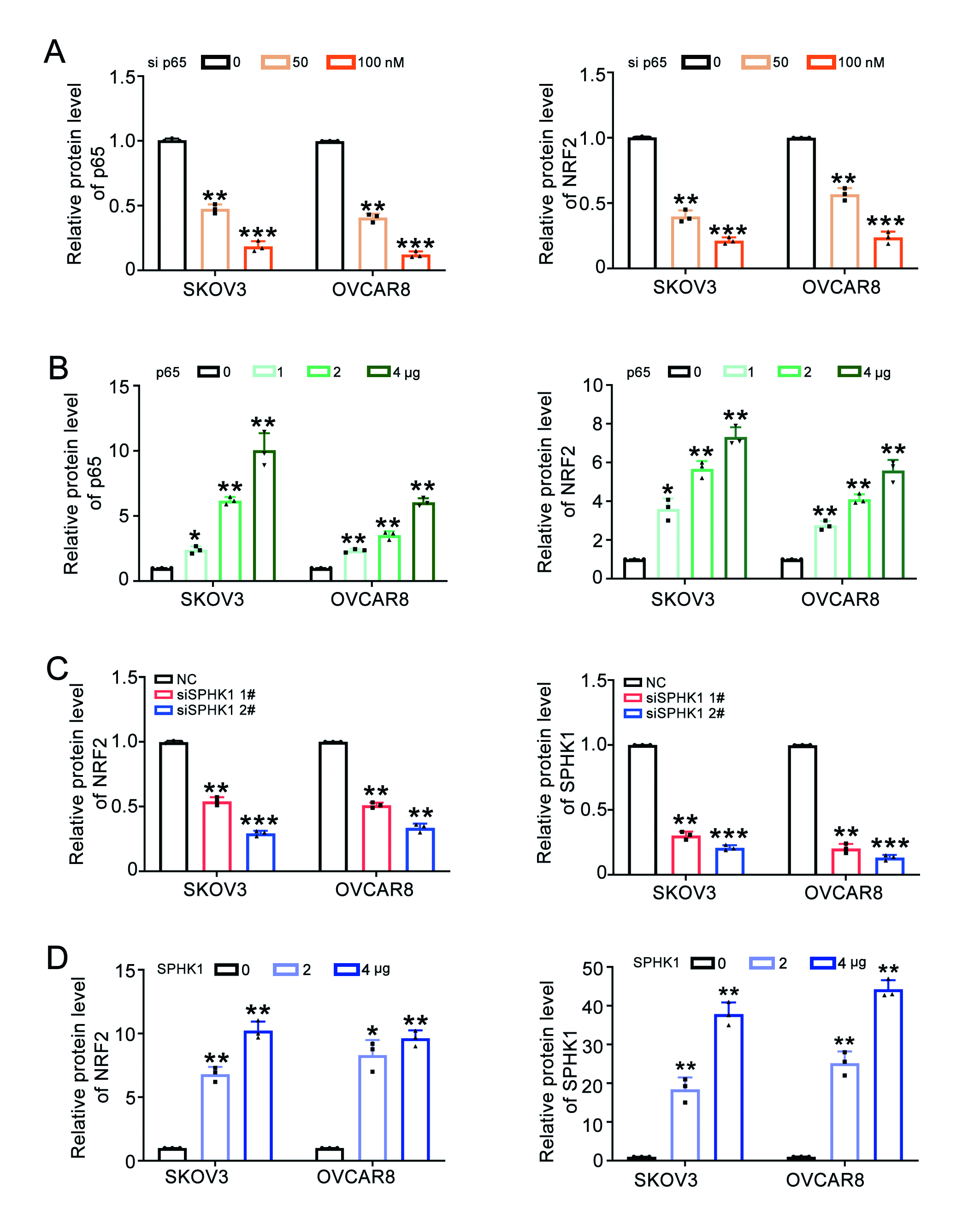
**

**Supplementary Figure 6**

**
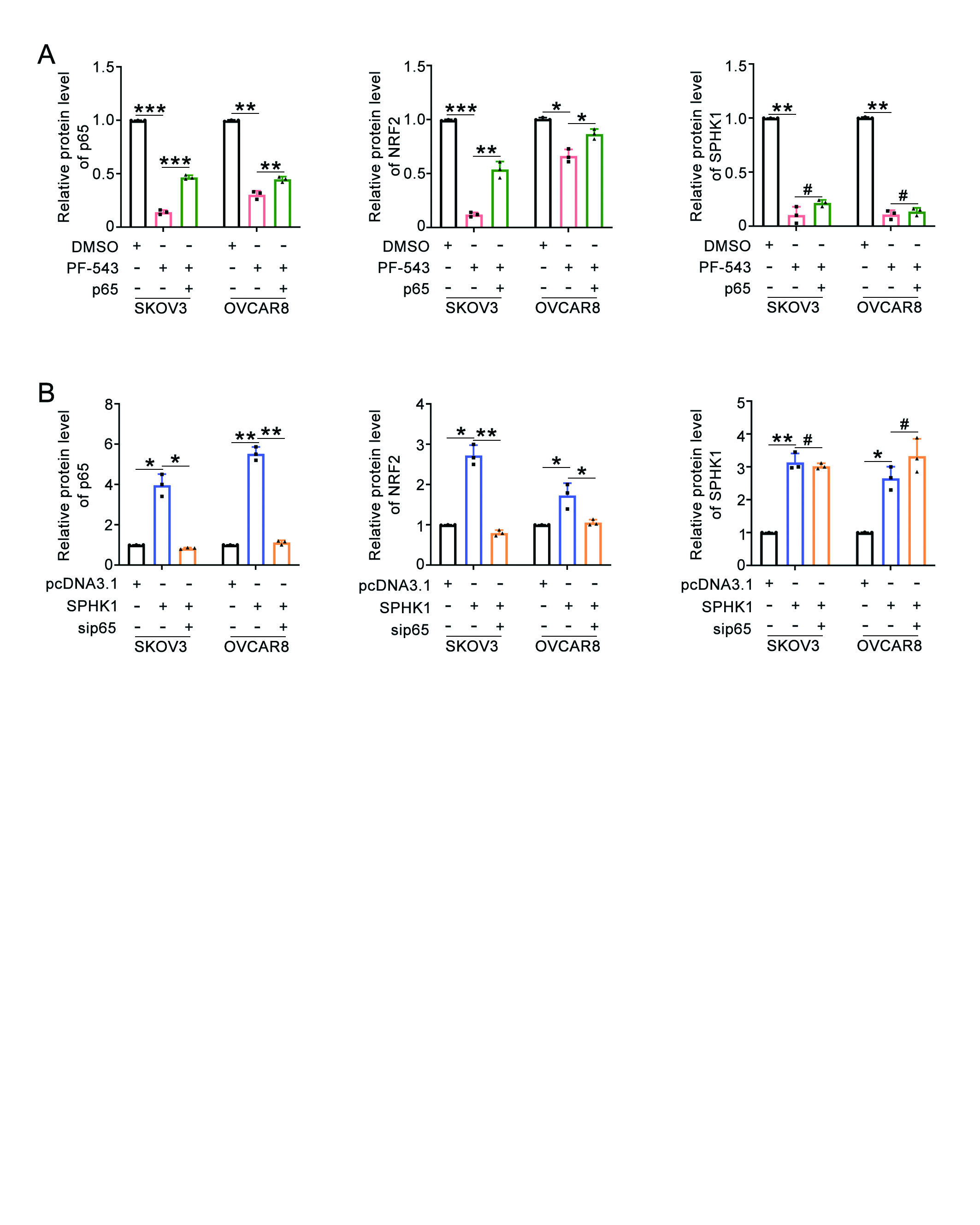
**
