# Supplementary material for: SPHK1 enhances olaparib resistance in ovarian cancer through the NFκB/NRF2/ferroptosis pathway
Source: Cell Death Discov. 2025 Jan 28;11:29. doi: 10.1038/s41420-025-02309-y (PMC11775125; doi:10.1038/s41420-025-02309-y)

**Original western blot Figures and figure legends**:

**Original western blot figure legends**:

**Original western blot Figure** **1. Original, full-length blot images of Figure 1 to Figure 4B.**

A. Original blot images of Figure 1G.

B. Original blot images of Figure 1H.

C. Original blot images of Figure 2B.

D. Original blot images of Figure 3B.

E. Original blot images of Figure 4A.

F. Original blot images of Figure 4B.

**Original western blot Figure 2.** **Original, full-length blot images of Figure 4C.**

1. Original, full-length blot images of Figure 4C.

**Original western blot Figure 3. Original, full-length blot images of Figure 4D-E.**

A. Original, full-length blot images of Figure 4D.

B. Original, full-length blot images of Figure 4E.

**Original western blot Figure 4. Original, full-length blot images of Figure 5A-B.**

A. Original, full-length blot images of Figure 5A.

B. Original, full-length blot images of Figure 5B.

**Original western blot Figure 5. Original, full-length blot images of Figure 7.**

A. Original, full-length blot images of Figure 7C.

B. Original, full-length blot images of Figure 7D.

C. Original, full-length blot images of Figure 7E.

D. Original, full-length blot images of Figure 7F.

E. Original, full-length blot images of Figure 7G.

F. Original, full-length blot images of Figure 7H.

**Original western blot Figures：**

**Original western blot Figure 1**

**
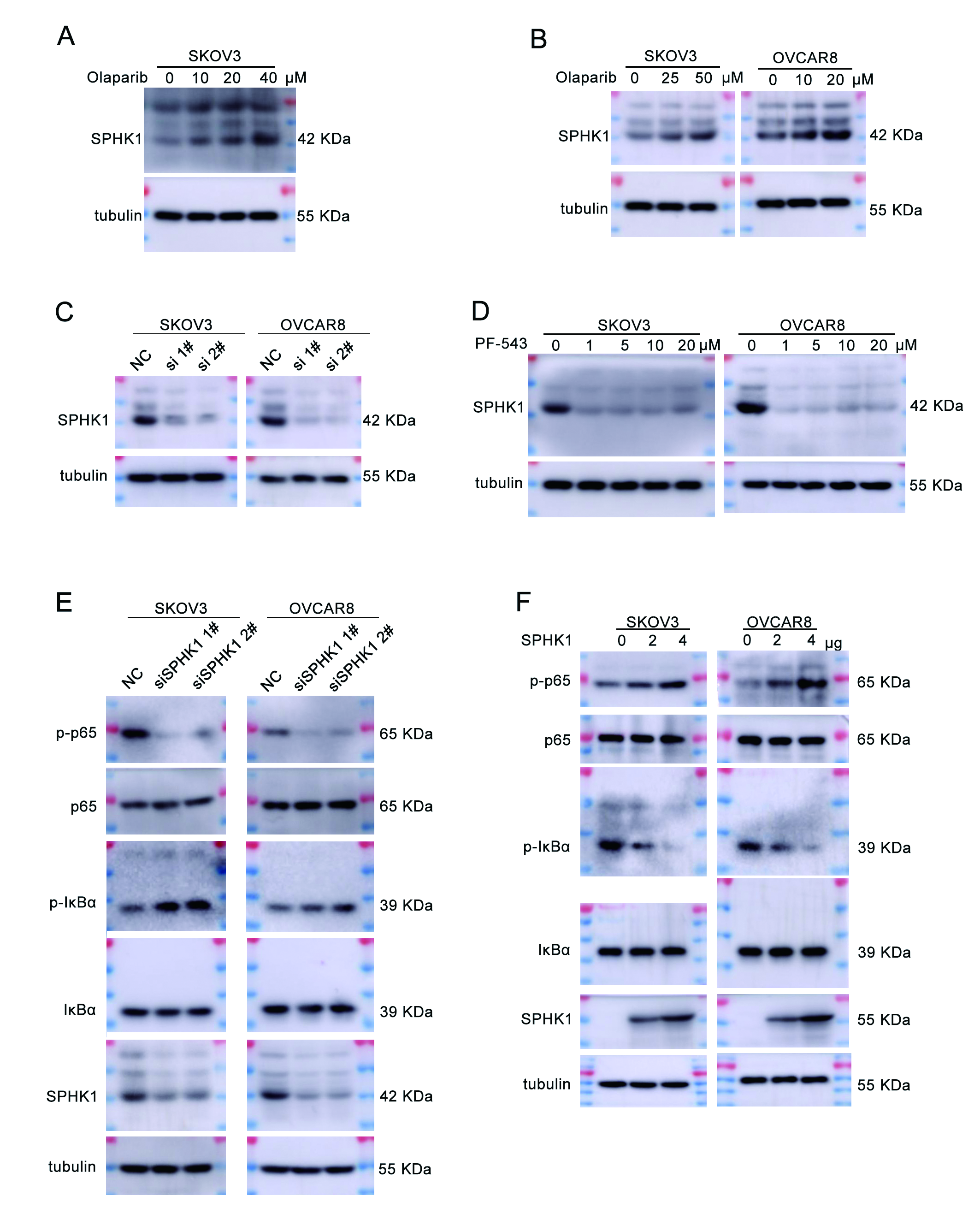
**

**Original western blot Figure 2**

**
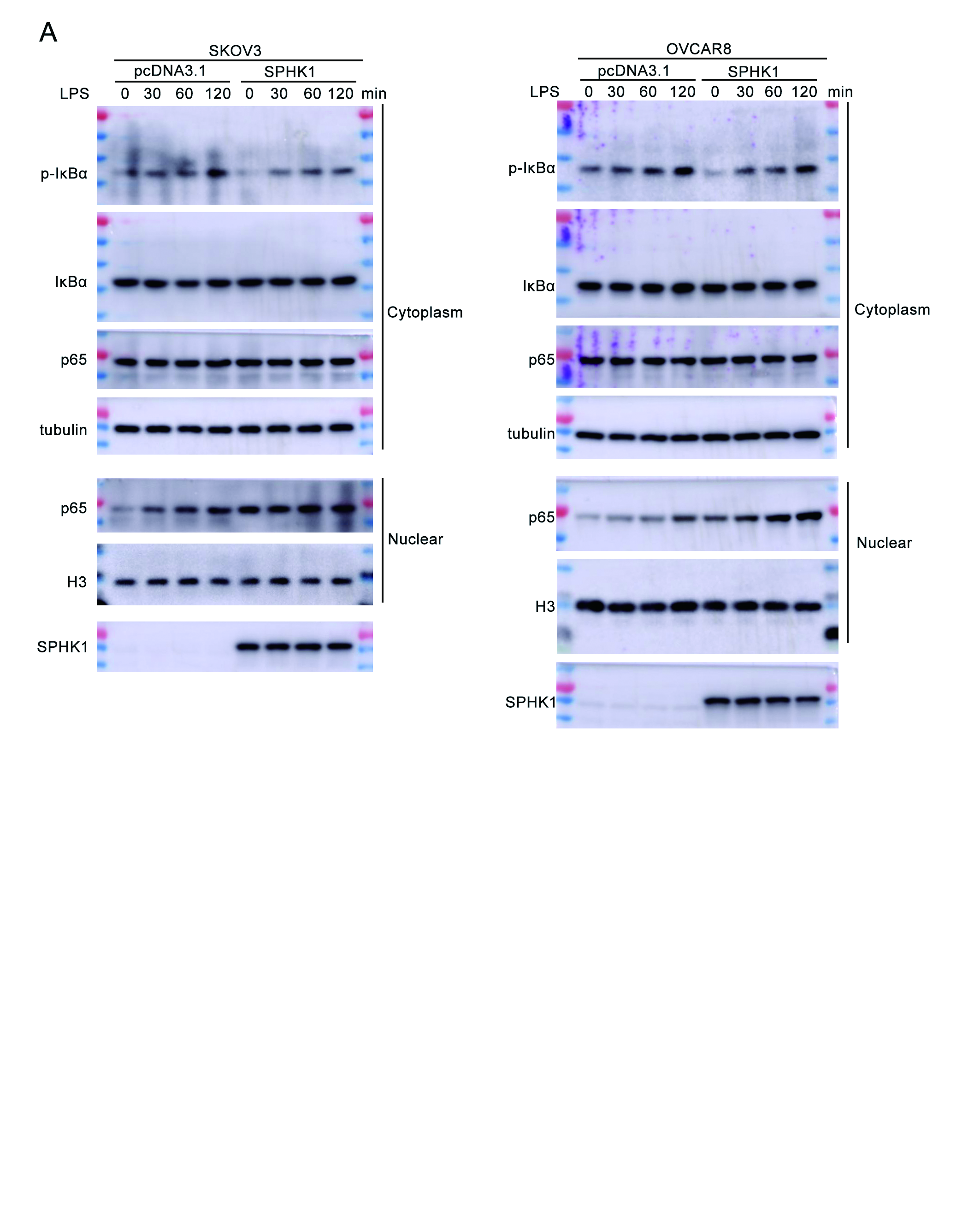
**

**Original western blot Figure 3**

**
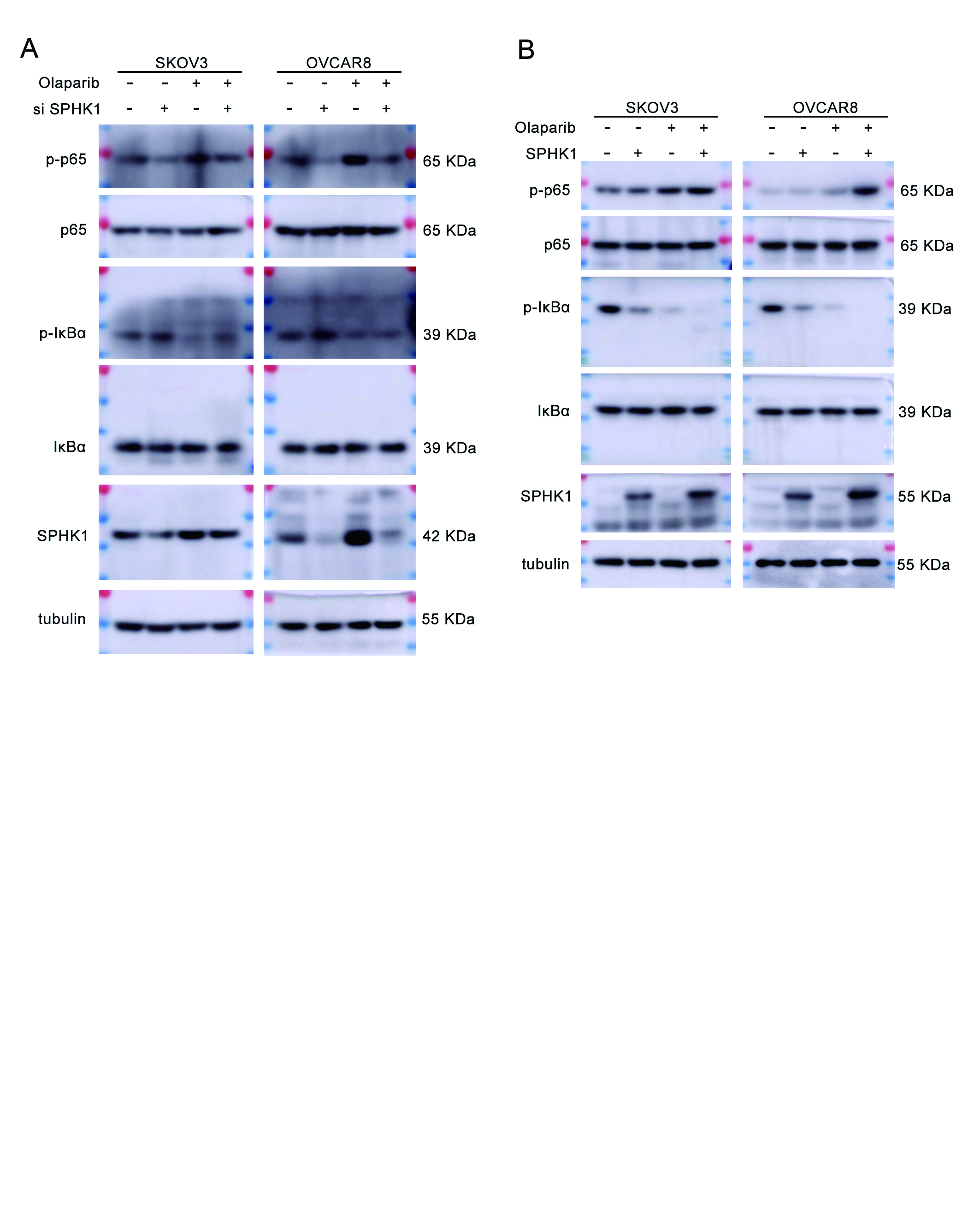
**

**Original western blot Figure 4**

**
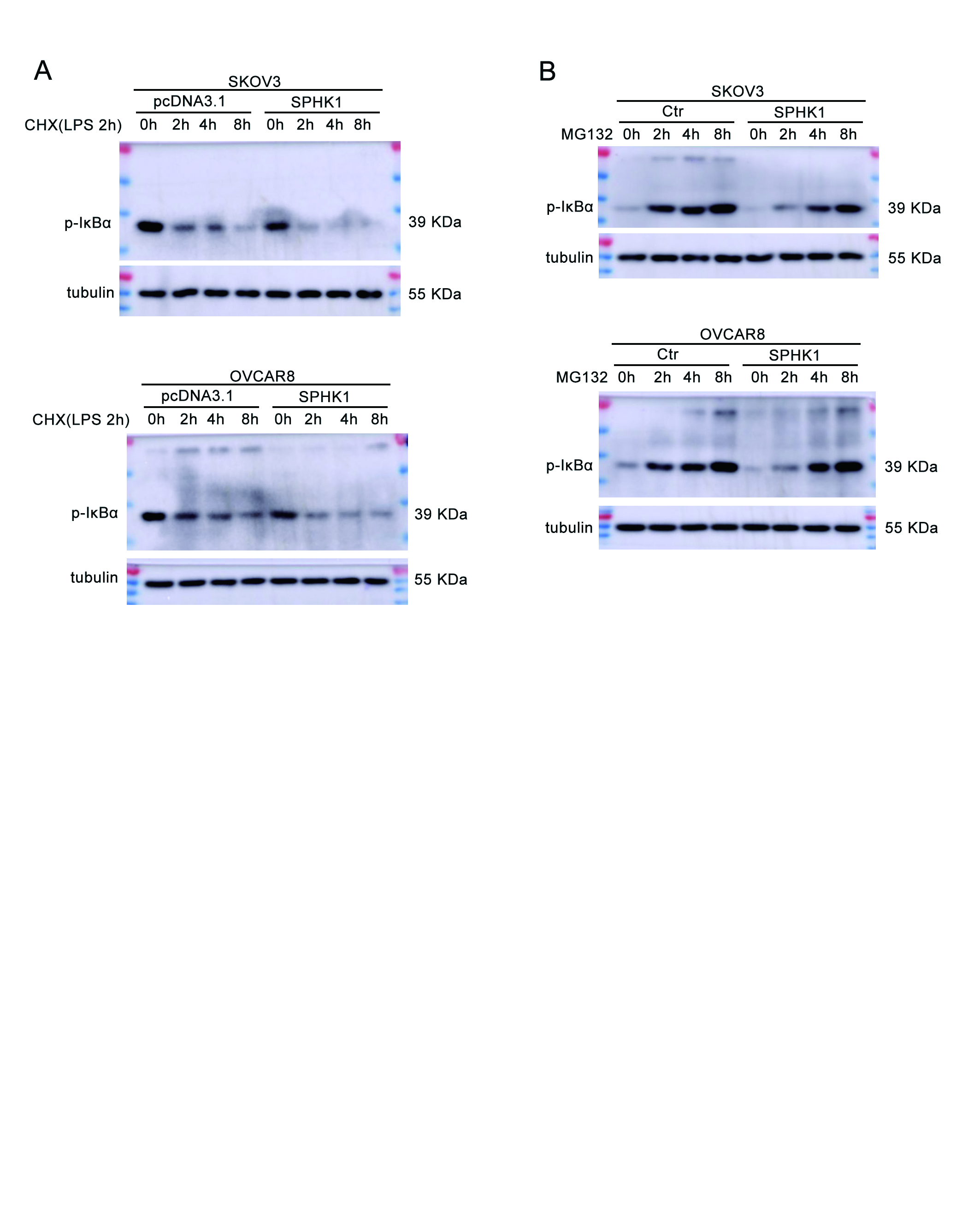
**

**Original western blot Figure 5**


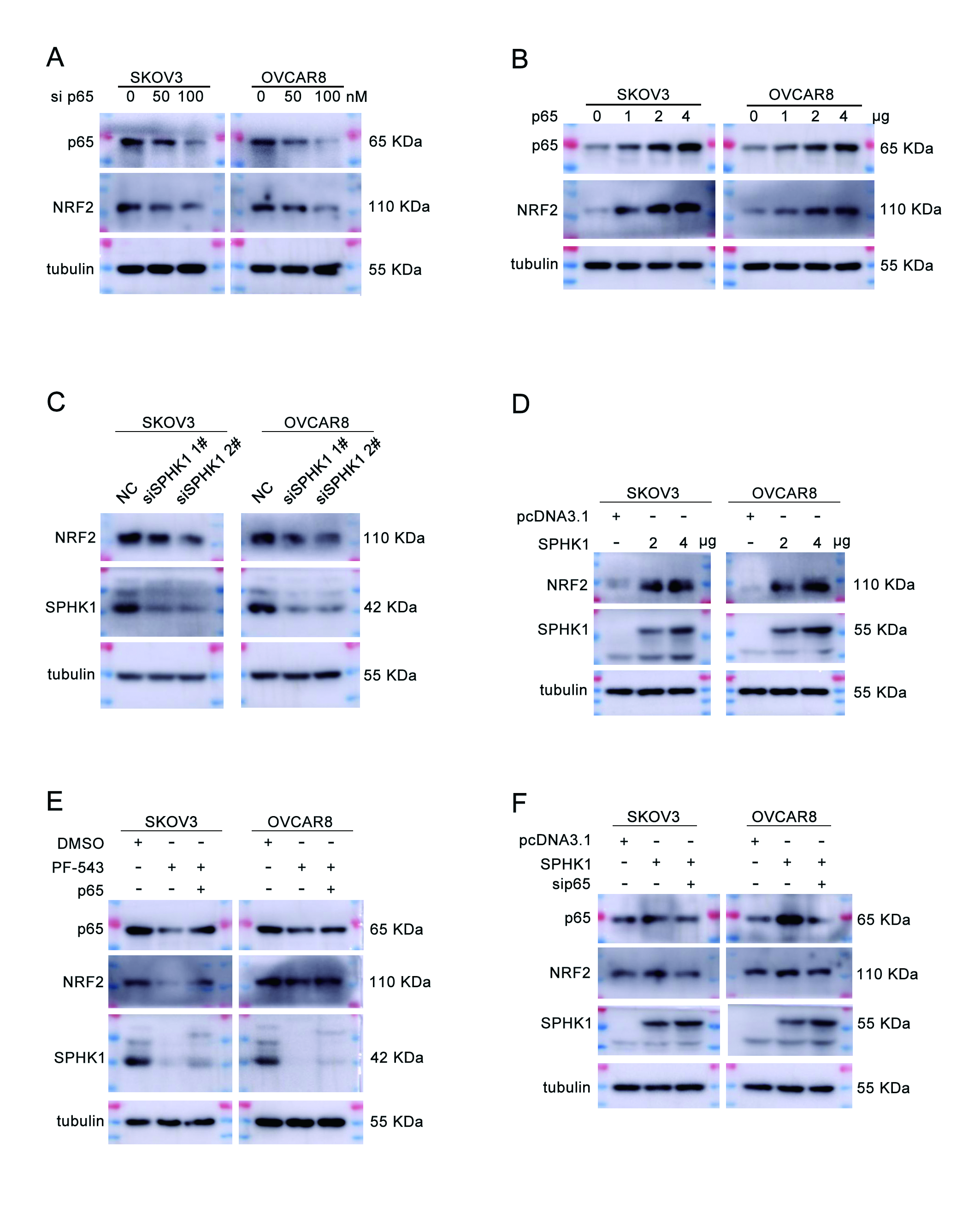

Supplement: Supplementary file 2 — Original figures of western blot [file 41420_2025_2309_MOESM2_ESM.docx]
